# Severe cutaneous anthrax with systemic complications: a case report

**DOI:** 10.3389/fmed.2026.1804212

**Published:** 2026-05-20

**Authors:** Uinkul Izbanova, Amangul Duisenova, Gulnara Tokmurziyeva, Aigul Abdrakhmanova, Saltanat Aibosynova, Bakhyt Kosherova, Ravilya Yegemberdiyeva, Ainur Sadykova, Saule Umarova, Daulet Askarov, Olzhas Rashkanov, Manshuk Sydykova, Aisazhan Yussupov, Altyn Rysbekova, Nur Tukhanova

**Affiliations:** 1 M.Aikimbayev’s National Scientific Center for Especially Dangerous Infections, Almaty, Kazakhstan; 2 Department Infectious and Tropical Disease, S.Asfendiyarov’s Kazakh National Medical University, Almaty, Kazakhstan; 3 I.Zhekenova’s City Clinical Infectious Disease Hospital, Almaty, Kazakhstan; 4 Karaganda Medical University, Karaganda, Kazakhstan

**Keywords:** cutaneous anthrax, endemic disease, One Health, secondary generalized anthrax, sepsis

## Abstract

In this case, we describe a severe case of human cutaneous anthrax complicated by secondary generalized form with bacteremia, sepsis, pulmonary involvement, and multiorgan dysfunction in an immunocompetent adult from Kazakhstan. A 31-year-old male presented with a progressive vesicles and bullae lesion on the right forearm associated with fever, extensive edema, following direct contact with a sick animal during slaughter. Wound exudate and blood sample demonstrated the presence of DNA *Bacillus anthracis* by PCR, and culture test of skin lesion confirmed the diagnosis. Despite early initiation of antimicrobial therapy, the patient developed sepsis, coagulation abnormalities, hepatic dysfunction, and bilateral pneumonia with pleural effusion, necessitating intensive care unit admission and escalation of antimicrobial and supportive treatment. Clinical and laboratory parameters gradually improved, and the patient recovered without late complications. This case highlights the potential for life-threatening systemic progression of cutaneous anthrax, emphasizes the diagnostic value of molecular detection of *B. anthracis* in blood, and underscores the importance of early recognition, close monitoring, and timely escalation of therapy in patients from endemic areas.

## 1 Introduction

Anthrax is an acute zoonotic infection caused by *Bacillus anthracis*, a spore-forming Gram-positive bacteria that primarily affects herbivorous animals and may be transmitted to humans through direct contact with infected animals, animal products, or contaminated environments (1, 2). Although the global incidence of anthrax has declined, the disease remains endemic in several regions, including Central Asia, Africa, and parts of the Middle East, where sporadic human cases continue to be reported (2, 3).

Cutaneous anthrax accounts for more than 95% of human anthrax cases and typically results from inoculation of spores through breaches in the skin (1, 3). The disease classically presents as a painless papule that progress to a vesicle and subsequently to a necrotic ulcer with a characteristic black eschar, often accompanied by surrounding edema and regional lymphadenitis. When promptly diagnosed and appropriately treated, cutaneous anthrax is generally associated with a favorable prognosis and low mortality (3, 4).

However, rare cases of cutaneous anthrax may progress to secondary generalized form, characterized by systemic dissemination of bacterial toxins (4, 5). This severe form may be complicated by sepsis, systemic inflammatory response syndrome, and multiorgan involvement, including pulmonary, hepatic, and hematological manifestations (5, 6). Published reports indicate that such progression is uncommon but associated with significantly increased morbidity and mortality, particularly in the setting of delayed diagnosis, high infectious inoculum, or inadequate initial therapy (4–7).

Laboratory confirmation is essential for the diagnosis of anthrax. While identification of *B. anthracis* from cutaneous lesions is typical in cutaneous anthrax, detection of the pathogen in blood is rare and usually indicates generalization of the process (3, 5). Molecular diagnostic methods, particularly polymerase chain reaction (PCR), play an increasingly important role in rapid confirmation, especially in severe cases where early diagnosis is critical for patient management (7).

Anthrax remains an important public health concern in Kazakhstan, where human cases continue to be reported annually, primarily associated with agricultural activities (8). In 2024, a total of 20 cases of human cutaneous anthrax were registered nationwide, corresponding to an incidence rate of 0.10 per 100,000 population. The majority of cases occurred in the Uygur District of the Almaty Region, followed by sporadic cases in the Zhambyl, Atyrau, and West Kazakhstan regions, as well as the city of Almaty (9). Epidemiological analysis demonstrated that the primary factor contributing to disease transmission was the slaughter of infected cattle, highlighting ongoing gaps in veterinary control and biosafety practices.

Here, we report a case of severe cutaneous anthrax complicated by secondary generalized form with bacteremia, sepsis, pulmonary involvement, and multiorgan dysfunction in an immunocompetent adult from an endemic region of Kazakhstan. This case highlights the potential for life-threatening progression of cutaneous anthrax and underscores the importance of early recognition, comprehensive diagnostic evaluation, and timely escalation of antimicrobial and supportive therapy.

## 2 Case presentation

**Figure 1:**
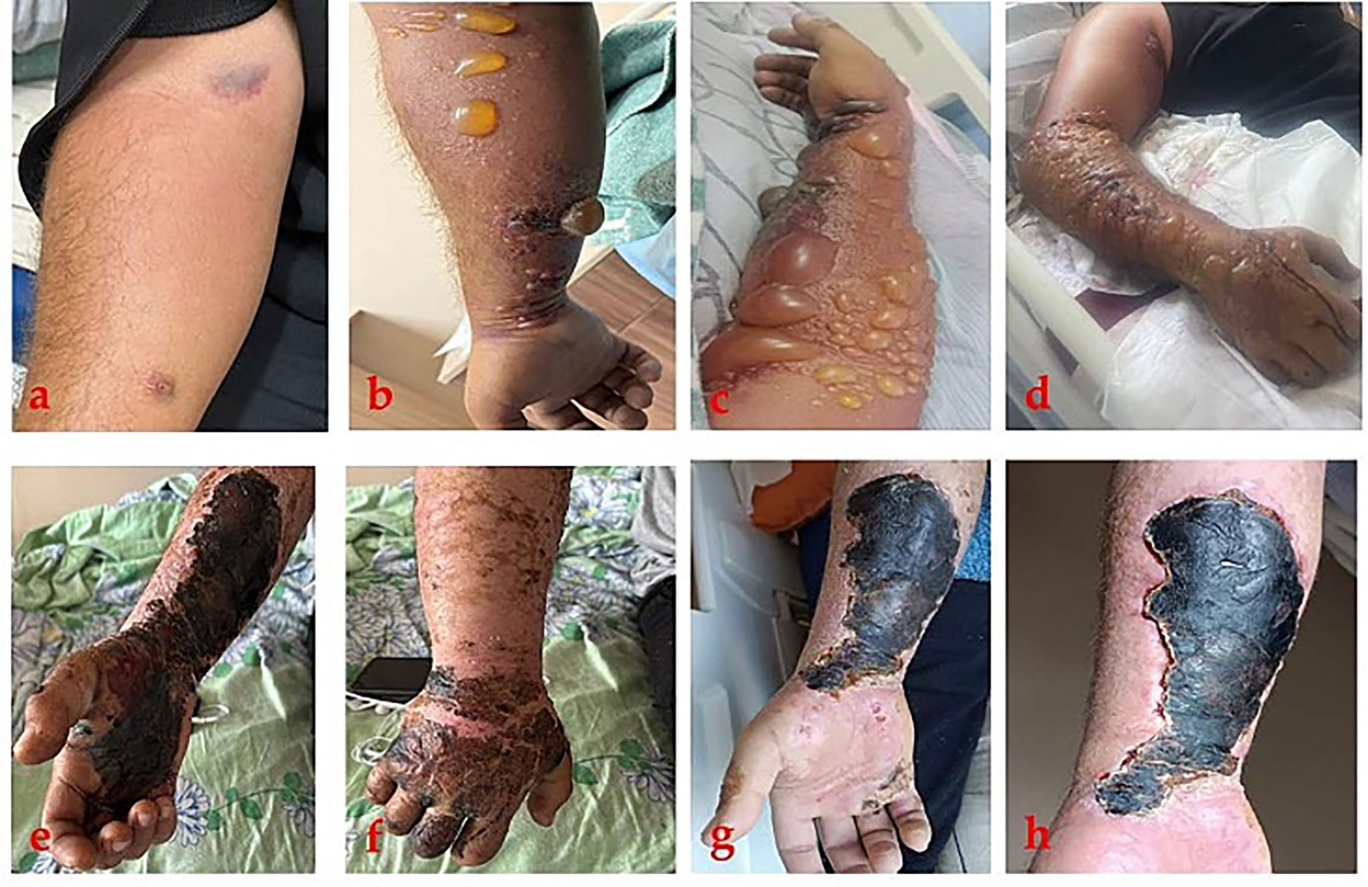
Clinical progression of cutaneous anthrax lesions on the right upper extremity: **(a)** Early stage 30.08: a small erythematous papule on the right forearm with minimal surrounding inflammation; **(b)** 01.09: formation of multiple vesicles and bullae with serous contents distant from the primary skin lesion with pronounced swelling of the forearm; **(c,d)** 06.09 progression characterized by extensive hemorrhagic and serous vesicles and bullae, widespread severe swelling of the forearm and wrist, in the area of the primary cutaneous affect hemorrhagic inflammation; **(e,f)** 01.10–10.10 on the inner side of the forearm and palm, and the back of the hand, black eschar (scab) formation with partial resolution of edema; **(g,h)** 23.10 black eschar (dry scab) sharply defined borders and surrounding granulation tissue.

A 31-year-old previously healthy male presented to the infectious disease emergency department with a multiple vesicles and bullae lesion on the right forearm accompanied by pain, fever, and generalized weakness. The disease had an acute onset on August 29, 2024, when a small painless papule appeared on the right forearm. On the following day, the patient developed fever up to 38 °C, generalized weakness, and myalgia (Figure 1a). By August 31, 2024, localized pain and progressive enlargement of the lesion were noted.

The patient was initially evaluated at the Uighur Central District Hospital and, due to persistent symptoms and lesion progression, was referred to the Almaty Multidisciplinary Clinical Hospital, followed by transfer to the Almaty City Infectious Diseases Hospital. His past medical history was unremarkable. He is a resident of the Almaty region (Uigur district, village of Shyryn) and works as a general laborer. On 25th of August 2024 he had participated, together with four other individuals, in the slaughter of sick cattle.

Upon admission on September 1, 2024, vital signs were as follows: temperature 37.8 °C, heart rate 89 beats/min, respiratory rate 17 breaths/min, blood pressure 100/80 mmHg, and oxygen saturation 96% on room air. Physical examination revealed multiple vesicles and bullae with serous contents on the medial surface of the right forearm around the primary lesion (~2 × 3 cm), surrounded by marked edema and a distinct inflammatory rim. Right axillary lymph node was enlarged, mildly tender, and not adherent to surrounding tissues (Figure 1b).

Initial laboratory investigations demonstrated mild leukocytosis (WBC 9.7 × 10^9^/L), neutrophilia (85.1%), lymphocytopenia (10%), elevated C-reactive protein (97.8 mg/L), mildly increased transaminases (ALT 84.9 U/L, AST 81.7 U/L), and slightly elevated procalcitonin (0.14 ng/mL). PCR testing of wound exudate and surrounding lesion smears, as well as blood samples, detected *Bacillus anthracis* DNA with detection of virulence-associated genes (*pag* and *cap*). Culture-based identification of wound material using standard bacteriological methods revealed non-hemolytic, Gram-positive bacilli with morphology consistent with *Bacillus anthracis*. Capsule formation was confirmed, and isolates were non-motile and susceptible to gamma phage lysis.

Antimicrobial susceptibility testing demonstrated susceptibility to all tested antibiotics, with intermediate susceptibility to erythromycin (Supplementary material 1). Whole genome sequencing confirmed the isolate as *Bacillus anthracis* with 100% reference similarity. Both virulence plasmids (pXO1 and pXO2) and key toxin and capsule genes were identified, consistent with a fully virulent strain.

In accordance with national surveillance procedures, clinical specimens were sent to the regional branch of the National Center for Expertise for initial PCR and bacteriological testing in the laboratory of particularly dangerous infections. After preliminary confirmation, the samples were forwarded to the M. Aikimbayev’s National Scientific Center for Particularly Dangerous Infections for confirmatory analysis.

Empirical antimicrobial therapy with ceftriaxone (4 g per day, IV in divided doses) and ciprofloxacin (200 mg per day IV in divided doses) intravenously was initiated.

On September 6, 2024, the patient’s condition deteriorated due to persistent fever and rapid progression of cutaneous lesions (Figures 1c,d). Laboratory findings revealed leukocytosis with neutrophilia, thrombocytopenia, markedly elevated inflammatory markers (CRP 200 mg/L, procalcitonin 4.25 ng/mL), significant coagulation abnormalities with elevated D-dimer levels (13.200 ng/mL (FEU)), and worsening hepatic dysfunction (ALT 107 U/L, AST 218 U/L) (Table 1). Bilateral pneumonia with right-sided pleural effusion was diagnosed based on clinical assessment and imaging during hospitalization (Supplementary material 2).

**Table 1:** Selected laboratory findings during hospitalization.

Parameter	At admission	Peak severity (ICU)^1^	At clinical improvement	Reference range
Hemoglobin (g/L)	161	78	133	120–160
Red blood cell count (×10^12^/L)	5.8	4.6	4.4	4.3–5.9
White blood cell count (×10^9^/L)	9.7	14.1	6.4	4.0–9.0
Neutrophils (%)	78.4	86.2	39.5	40–74
Platelets (×10^9^/L)	186	127	289	180–320
C-reactive protein (mg/L)	97.8	200	22	<5
Procalcitonin (ng/mL)	0.9	4.25	0.28	0.02–0.10
D-dimer (ng/mL)	2,480	13,200	245	< 500 ng/mL (FEU)
Alanine aminotransferase ALT (U/L)	84.9	107	41	<40
Aspartate aminotransferase AST (U/L)	81.7	218	46	<40
Albumin (g/L)	38	33	41	35–52

^1^ICU-intensive care unit. Values represent clinically relevant time points rather than daily measurements.

Given the development of sepsis and respiratory involvement, the patient was transferred to the intensive care unit, where he remained from September 6 to September 16, 2024. Antimicrobial therapy was intensified by replacing ceftriaxone with meropenem (4 g per day IV twice daily), increasing dose of ciprofloxacin (400 mg IV twice daily) and adding amikacin (1.5 g per day, IM in three divided doses). Supportive management in the intensive care unit included oxygen therapy delivered via nasal cannula, intravenous fluid therapy for hemodynamic stabilization, and continuous monitoring of coagulation parameters due to markedly elevated D-dimer levels.

During the intensive care unit stay, gradual clinical stabilization was achieved, with resolution of fever, reduction of limb edema, and improvement of systemic inflammatory markers. The patient was transferred from the intensive care unit to the department; antibacterial therapy was changed to doxycycline (200 mg per day) for 2 weeks and supportive therapy was continued. Over the subsequent weeks, the cutaneous lesions evolved through eschar formation and partial resolution of edema (Figures 1e,f). Systemic manifestations resolved completely, laboratory parameters normalized, and radiological signs of pneumonia regressed.

The patient completed antibacterial treatment and was discharged on October 23, 2024, in stable condition, with residual black eschar sharply defined borders and surrounding granulation tissue (Figures 1g,h) for home isolation and further observation at the outpatient. We did not find out the patient’s follow-up information, but information from outpatient revealed that the patients lesion healed by December.

## 3 Discussion

The present case illustrates an uncommon but clinically significant progression from localized cutaneous disease to severe secondary generalized form in an immunocompetent adult, emphasizing that cutaneous anthrax cannot invariably be regarded as a self-limited infection.

The patient had a clear epidemiological risk factor, direct contact with a sick animal during slaughter in an endemic rural area, which remains the predominant route of transmission for cutaneous anthrax worldwide (10–12). Similar exposures have been consistently identified as major risk factors in endemic regions, underscoring the persistent public health relevance of anthrax in agricultural communities despite global declines in incidence (13).

Early clinical recognition of cutaneous anthrax remains essential, particularly in endemic regions. In the present case, two early clinical indicators were particularly suggestive of anthrax infection: the appearance of a painless papule rapidly progressing to vesicles and bullae with marked surrounding edema, and a clear epidemiological exposure history involving direct contact with sick livestock during slaughter. The combination of characteristic lesion evolution and epidemiological risk factors should prompt immediate clinical suspicion and early microbiological investigation.

The pronounced local manifestations observed in this patient, including extensive edema, vesiculation, hemorrhagic bullae, and progressive tissue necrosis, are consistent with the known pathophysiological effects of anthrax toxins. Edema toxin and lethal toxin disrupt endothelial integrity, increase vascular permeability, and impair host immune responses, leading to both severe local tissue injury and systemic inflammatory effects (14). In the present case, these local findings were accompanied by a systemic inflammatory response, thrombocytopenia, and coagulation abnormalities, consistent with sepsis and toxin-mediated endothelial dysfunction and evolving sepsis (15).

Pulmonary involvement represented a major complication and an important determinant of disease severity in this patient. Respiratory manifestations, including pneumonia and pleural effusion, are classically associated with inhalational anthrax and are considered markers of poor prognosis (5). However, pulmonary complications have also been described in severe systemic forms of cutaneous anthrax, likely reflecting hematogenous dissemination of bacteria or toxins, endothelial injury, and secondary inflammatory processes (11, 15). The development of bilateral pneumonia with pleural effusion in this case necessitated intensive care unit admission and underscores the need to consider respiratory involvement as a potential complication of generalized cutaneous anthrax.

Hepatic dysfunction observed during the acute phase further supports the diagnosis of multiorgan involvement. Liver injury in severe anthrax has been attributed to a combination of systemic inflammation, hypoperfusion, hypoxia, and the direct toxic effects of bacterial products (2, 12). Similar hepatic abnormalities have been reported in anthrax-related sepsis and are regarded as indicators of severe systemic disease rather than primary hepatic infection (10). In the present patient, hepatic dysfunction was supported by significant elevations of alanine aminotransferase (ALT) and aspartate aminotransferase (AST) during the peak phase of systemic illness, which subsequently normalized following clinical stabilization.

Laboratory confirmation of systemic dissemination represents a particularly important aspect of this case. Detection of *B. anthracis* in blood is uncommon in cutaneous anthrax and typically indicates advanced disease with a high risk of adverse outcomes (4, 10). Molecular detection of *B. anthracis* DNA in blood, as observed in this patient, has been reported only sporadically in the literature and is usually associated with severe clinical manifestations, including sepsis and multiorgan involvement (16, 17).

Previous reports have also indicated that inappropriate local manipulation of anthrax lesions, including surgical incision, drainage, or traditional scarification practices, may increase the risk of systemic dissemination by disrupting local tissue barriers and facilitating hematogenous spread of the pathogen. Importantly, in the present case no surgical incision, drainage, or invasive manipulation of the lesion was performed prior to clinical deterioration, suggesting that systemic progression may occur even in the absence of such aggravating factors (18, 19).

Recent reports of severe cutaneous anthrax published in recent years have described similar patterns of systemic inflammation, coagulopathy, and multiorgan dysfunction (6, 7). Compared with these reports, the present case demonstrated comparable laboratory abnormalities including elevated inflammatory markers, thrombocytopenia, and hepatic enzyme elevation. However, the simultaneous occurrence of bacteremia confirmed by PCR, bilateral pneumonia with pleural effusion, and marked coagulation abnormalities represents a relatively uncommon constellation of findings.

Antibacterial management of this patient evolved in response to disease severity and systemic complications. Initial empirical therapy with ceftriaxone and ciprofloxacin was appropriate for suspected cutaneous anthrax. It should be noted that third-generation cephalosporins, including ceftriaxone, are generally not recommended for the treatment of anthrax because *Bacillus anthracis* may exhibit intrinsic resistance mediated by constitutive cephalosporinases (6) however, in this case ceftriaxone was initiated empirically before microbiological confirmation as part of broad-spectrum therapy for suspected severe bacterial skin infection. Rapid progression to sepsis and respiratory involvement required escalation to combination therapy with meropenem, high-dose ciprofloxacin, and amikacin. This intensified regimen aligns with recommendations for severe anthrax with systemic manifestations and resulted in clinical and laboratory stabilization (2, 6). Following recovery, step-down therapy with oral doxycycline allowed completion of treatment with favorable outcome. Current international recommendations advocate fluoroquinolone-based combination therapy for severe anthrax, with intensive supportive care in cases complicated by sepsis or organ failure, as outlined by the World Health Organization and other expert bodies (2, 6). The need for escalation of antimicrobial therapy in this patient highlights the importance of dynamic reassessment and individualized treatment strategies in severe anthrax.

This case adds to the limited body of literature documenting secondary generalized disease arising from cutaneous anthrax and underscores several clinically relevant points. First, cutaneous anthrax may progress rapidly to life-threatening systemic disease, even in immunocompetent individuals. Second, pulmonary and hepatic involvement should be recognized as markers of severe systemic dissemination. Third, molecular detection of *B. anthracis* in blood may serve as an early indicator of poor prognosis and should prompt aggressive management. Finally, timely escalation of antimicrobial and supportive therapy can result in favorable outcomes, even in severe cases traditionally associated with high mortality.

## 4 Conclusion

This case demonstrates that cutaneous anthrax may progress to secondary generalized disease with bacteremia, sepsis, and multiorgan involvement, even in immunocompetent individuals. Early recognition of systemic dissemination, prompt laboratory confirmation, and timely escalation of antimicrobial and supportive therapy are critical to achieving favorable outcomes in severe cases.

## Data Availability

The raw data supporting the conclusions of this article will be made available by the authors, without undue reservation.
