# Supplementary material for: Severe cutaneous anthrax with systemic complications: a case report
Source: Front Med (Lausanne). 2026 May 20;13:1804212. doi: 10.3389/fmed.2026.1804212 (PMC13229773; doi:10.3389/fmed.2026.1804212)
Supplement: Supplementary file 1 [file Data_Sheet_1.PDF]

Results of the antibiotic susceptibility test using the disk method

Patient	ID	Antibiotics										
		Ceftriaxone (30 µg)	Kanamycin (30 µg)	Rifampicin (5 µg)	Gentamicin (10 µg)	Erythromycin (15 µg)	Tetracycline (30 µg)	Ampicillin (10 µg)	Ciprofloxacin (5 µg)	Streptomycin (10 µg)	Doxycycline (30 µg)	Meropenem (10 µg)
		Susceptibility Breakpoint (Zone Diameter, mm)										
		≥29	≥15	≥18	≥15	≥23	≥19	≥21	≥21	≥15	≥23	≥26
Patient 1	*B. ant.* KZ-Alm-1-2024	31,4 S	22.0 S	21.1 S	29.2 S	15.8 I	31.6 S	24.9 S	30.6 S	25.4 S	34.2 S	32.2 S
Patient 1	*B. ant.* KZ-Alm-2-2024	30,6 S	24.5 S	21.0 S	23.8 S	19.9 I	31.1 S	30.4 S	33.2 S	21.3 S	36.5 S	30.2 S
