# Supplementary material for: Severe cutaneous anthrax with systemic complications: a case report
Source: Front Med (Lausanne). 2026 May 20;13:1804212. doi: 10.3389/fmed.2026.1804212 (PMC13229773; doi:10.3389/fmed.2026.1804212)
Supplement: Supplementary file 2 [file Data_Sheet_2.PDF]

Қазақстан Республикасы<br>Денсаулық сақтау министрлігі<br>Министерство здравоохранения<br>Республики Казахстан		ҚҰЖЖ бойынша ұйым коды 39002104<br>Код организации по ОКПО 39002104
Ұйымның атауы<br>Наименование организации:<br>Городская клиническая инфекционная больница им. И.С. Жекеновой<br>Республика Казахстан, г. Алматы, ул. Дегдар 10/2		Қазақстан Республикасы<br>Денсаулық сақтау министрінің м. а. 2020 жылы «30» қазан № ҚР ДСМ-175/2020<br>бұйрығымен бекітілген Медициналық құжаттама
		Медицинская документация<br>Утверждена Приказом<br>и.о. Министра здравоохраненияРеспублики Казахстан<br>от 30 октября 2020 года № ҚР ДСМ-175/2020.

Результаты исследований - Рентгенография обзорная органов грудной клетки (1 проекция)

Двухсторонняя пневмония. Правосторонний плеврит.

Chest radiography (single projection): Bilateral pneumonia. Right-sided pleuritis.

Талдау жасалған күні және уақыты
(Дата и время выполнения): **06.09.2024 09:05**
